# Association of Cognitive Abilities and Brain Lateralization among Primary School Children in Kuwait

**DOI:** 10.1155/2016/6740267

**Published:** 2016-05-26

**Authors:** Jasem Y. Al-Hashel, Samar Farouk Ahmed, Hanouf Al-Mutairi, Shahd Hassan, Nora Al-Awadhi, Mariam Al-Saraji

**Affiliations:** ^1^Department of Neurology, Ibn Sina Hospital, P.O. Box 25427, 13115 Safat, Kuwait; ^2^Department of Medicine, Faculty of Medicine, Kuwait University, P.O. Box 24923, 13110 Safat, Kuwait; ^3^Department of Neurology and Psychiatry, Minia University, P.O. Box 61519, Minia 61111, Egypt; ^4^Kuwait Institute for Medical Specialization, P.O. Box 1793, 13018 Safat, Kuwait; ^5^Kuwait Center for Mental Health, P.O. Box 4081, 13041 Kuwait City, Kuwait; ^6^Department of Medicine, Mubarak Al-Kabeer Hospital, P.O. Box 24923, 13110 Safat, Kuwait

## Abstract

*Background*. Many studies have explored the cognitive variation between left- and right-handed individuals; however, the differences remain poorly understood.* Aim of the Work*. To assess the association between brain lateralization indicated by handedness and cognitive abilities.* Material and Methods*. A total of 217 students aged between 7 and 10 years of both genders were identified for the study. Males and females were equally distributed. All left-handed students were chosen. An equal group with right-handed students was randomly selected. Handedness was assessed using traditional writing hand approach as well as the WatHand Cabient Test and the Grooved Pegboard Test. Cognition was measured using Cambridge University's CANTAB eclipse cognitive battery. Pearson Correlation Coefficient Test “*r*” was calculated to measure the strength of association between quantitative data.* Results*. Right-handed children had superior visuospatial abilities (*p* = 0.011, *r* = 0.253), visual memory (*p* = 0.034, *r* = 0.205), and better scores in reaction time tests which incorporated elements of visual memory (*p* = 0.004, *r* = −0.271). Left-handed children proved to have better simple reaction times (*p* = 0.036, *r* = 0.201).* Conclusion*. Right-handed children had superior visuospatial abilities and left-handed children have better simple reaction times.

## 1. Introduction

Handedness refers to the preference for using right or left hand for various unimanual activities [1]. Many methods are used to determine handedness including self-reported questionnaires, dexterity tasks, and grip strength [2]. The proportion of left- and right-handed varies significantly from study to study and among different races. This might be attributed to different incidence of the two categories of handedness, cultural differences in acceptance of left hand preference in different populations, heterogeneity of handedness phenomena, and discrepancy of methods used for assessment of handedness [3–6].

Handedness is known to be related to dominant hemisphere [7] and these asymmetries are fundamental to human cognition [8]. A better understanding of the effect of handedness on child cognitive development will help to explain any observed educational differentials. Several cognitive indices such as reaction time and executive function were examined to compare right and left-handed individuals. Some studies suggest that there is an inherent advantage to being left-handed in reaction time [9]. In terms of executive function, a correlation has been observed between handedness and visuospatial processing. Left-handed individuals perform better than right-handed ones [10].

The issue of selection of behavioral indexes of brain lateralization is very complex. Eye, ears, and foot are important in the consideration of cerebral specialization. Although different experimental work has attempted to identify reliable behavioral predictor of cerebral lateralization, no such predictor has been found. Previous study demonstrated that footedness is a possible indicator of human laterality [11]. Preferred handedness has received the most experimental attention.

We aimed to assess the association between brain lateralization and cognition as handedness was used as an index of brain lateralization.

## 2. Material and Methods

There is currently no data reflecting the prevalence of right- and left-handedness in Kuwait. In order to ensure adequate representation of both right- and left-handed subjects, an equal sample of both right- and left-handed subjects was sought.

We chose a young population as evidence suggesting that the prevalence of left-handedness declines with increasing age [12]. Schools provided a convenient population as the Ministry of Education oversees the public schools in each governorate via six educational areas and enforces a uniform curriculum and assessment method thereby making children attending public schools an ideal study population. The tools we used to assess cognition are designed for children aged six and above. As first graders in Kuwait can be as young as five years old, we chose school children of both genders attending public school from second to fifth grade as our sample. In order to select a socioeconomically representative sample of children, two public schools were randomly chosen from each of Kuwait's six governorates. As schools are gender segregated in Kuwait, we chose one boys' school and one girls' school from each governorate for a total of twelve schools, with an additional twelve back up schools. Schools were selected by assigning a number to each school and using a random number generator.

Subjects for the study were selected from each school by visiting classes from each grade. Left-handed students were identified by either asking the students to raise the hand which they use to write or asking them to write their names or draw a check mark to observe their hand of choice. All left-handed students identified by these methods were included in the study.

In order to ensure adequate representation of both right- and left-handed subjects, an equal sample of both right- and left-handed subjects was sought. We included right- and left-handed groups according to the values of laterality indexes.

All selected students were given informed consent and self-administered questionnaires to take home to their guardians and were instructed to return signed consent and the filled out questionnaires and leave them with the school social worker for safekeeping until our return to the school for data collection two days later. Children who returned completed questionnaires were assigned to one of two sets of serial numbers reflecting their writing hand preference; within each set, students had equal opportunity of having odd and even serial numbers which aided in further randomization. The questionnaire consisted of questions regarding the child's personal information and information regarding the family's sociodemographic status. It is possible that left-handed children have early been corrected to being right-handed, so the questionnaire included questions to parents to clarify which preferred hand is since birth and if being left-handed has been corrected to being right-handed.

Handedness was assessed by two tests: the Grooved Pegboard Test (GPT) and the WatHand Cabient Test (WBT) [13–15]. GPT compares the speed with which children are able to manipulate, place, and remove metal pegs with keys on one side from randomly positioned slots on a pegboard using their right and left hands independently. Two trials were undergone for each hand; students with even serial numbers commenced with the left hand and students with odd serial numbers with the right one. The WBT counts the frequency of right and left hand used in performing various manual tasks, including opening a door, using a hammer and screwdriver, pressing a button, hanging a washer on a hook, opening a lock with a key, retrieving candy from a candy dispenser, and throwing a ball at a target. Each task was performed twice in no particular order. Both the GPT and the WBT provided us with laterality indices calculated using the following equation (*R* − *L*)/(*R* + *L*) producing a continuous scale in which 1 and −1 represent absolute right- and left-handedness, respectively.

Cognition was assessed with The Cambridge Neuropsychological Test Automated Battery (CANTAB) which is a group of computer-based tests that assess visual memory, attention, and executive function [16].

CANTAB is a computerized neuropsychological assessment battery originally written and developed by Barbara Sahakian, Trevor Robbins, and colleagues at Cambridge University in 1986 [16]. It has been used in a wide variety of clinical populations [16, 17] with different levels of ability and ages. It has also been employed in neuropsychological research across age groups to study the development of a set of cognitive functions [18].

Visual memory was assessed with the Delayed Matching to Sample (DMS) test, Pattern Recognition Memory (PRM) test, and Spatial Recognition Memory (SRM) test. In the DMS test, the subject must memorize a visual pattern and then recognize it from a group of similar patterns after various delay intervals. In the first phase of the PRM test, the subject is shown a series of 12 patterns. In the second phase, the subject must choose from two patterns presented at a time, one of which has already been shown and the other is a novel distractor. The SRM test assesses the subject's spatial recognition by first displaying a series of white boxes in different locations. These boxes reappear one at a time along with a new box, and the subject is requested to recognize the familiar ones.

The executive function test used is the Spatial Span (SSP) that assesses working memory in addition to visuospatial ability. Nine white squares appear on the screen, some of which change color in a sequence, and the subject must choose these squares in the same order. The sequence of squares starts with two and increases to a maximum of nine squares with every correct answer.

The remaining two tests assess attention and require the use of a two-button press pad. In the (CRT) test, subjects are presented with a series of arrows located in either the right or left half of the screen and are required to press the corresponding right or left button on the press pad.

In the Reaction Time (RTI) test, the subject is required to press a button on the press pad with the index finger until a circle appears on the screen. The subject must then react by immediately touching that circle with the same finger. The test is divided into two phases; in the first phase, the circle always appears in the center of the screen, while in the second, it may appear in one of five predefined locations.

In order to exclude children who are unable to comprehend commands or have visual or movement difficulties, all subjects began their CANTAB testing with the Motor Screening Task (MOT), a test in which the subject is required to touch a flashing cross that appears in different locations on the screen.

All tests were carried out in school libraries that provided a quiet, distraction-free environment for the students. Additionally, all students wore noise canceling headphones for the duration of the CANTAB tests to enhance the audio component of the tests and facilitate the test in a friendly environment. Ethical approval for this study was obtained from the Ministry of Education that oversees all the public schools from which the students were sampled.

Statistical analysis was performed using the Statistical Package for Social Sciences (SPSS). Pearson Correlation Coefficient Test “*r*” was calculated to measure the strength of association between values of laterality indexes and cognitive functions. Statistical significance was defined as a *p* value < 0.05.

## 3. Results

Out of 410 questionnaires that were distributed, 233 were completed and returned (57%). The remaining 43% represented those students who were absent or failed to deliver the questionnaires due to either negligence or refusal of the parents to participate.

Fifteen students were excluded on account of not meeting the age criteria at the time of the test. Ten patients were excluded because they were unable to comprehend commands or have visual or movement difficulties. The effective sample size was 217 students (110 right-handed and 107 left-handed) ranging from the ages of seven to ten. Males and females were equally distributed, 108 males versus 109 females. Students aged nine comprised 31.1% with the remaining ages each not exceeding one fourth of the sample. All age groups had a difference of less than 10% in terms of distribution among right- and left-handed students (according to preferred writing hand) except for those aged 8 years where the majority was left-handed (63%). The majority of the students (56.8%) were female with near equal representation of students writing with their left hand between both genders.

The three measures of direction of handedness were compared using two-tailed Spearman correlation. A statistically significant correlation was found between all three measures, *p* = 0.01. The strongest correlation was found between WBT and the writing hand, *r* = 0.6 (Table 1).

There was no statistically significant correlation between any of the three tests of handedness and the parameters of the CRT test. The DMS test was significantly correlated in the parameters of mean correct latency (in which the triggers were not hidden) with the WBT (*p* = 0.004, *r* = −0.271) (Table 2).

With regards to the PRM test, there was a significant correlation in the percent of correct answers with both the GPT (*p* = 0.034, *r* = 0.205) and the writing hand (*p* = 0.025, *r* = 0.216) (Table 3). The RTI test showed a significant correlation between the mean simple reaction time and the GPT (*p* = 0.036, *r* = 0.201) (Table 4). The SSP demonstrated a significant correlation between the maximum length of the sequence reached and the GPT (*p* = 0.011, *r* = 0.253) as well as between the total number of errors and the GPT (*p* = 0.02, *r* = 0.228); the total usage errors (i.e., errors committed by selecting a box not in the original sequence) were also significantly correlated with the WBT (*p* = 0.016, *r* = −0.235) (Table 5).

Factors that were not significantly associated with cognition include gender and parental marital status and their educational level.

## 4. Discussion

Selection of behavioral indexes of brain lateralization is very complex. Preferred hand, eye, ears, and foot are important in the consideration of cerebral specialization. Preferred handedness has been studied in many previous studies [11]. Our cohort included children of age 7–10 years and we conducted the study at schools during the ordinary days. It is very difficult to assess other predictors of cerebral laterization.

Our study included 217 students of ages ranging from seven to ten. Males and females were equally distributed with near equal representation of students writing with their left hand between both genders.

Our results showed that right-handed children had significant superior visuospatial abilities, visual memory, and better scores in reaction time tests which incorporated elements of visual memory. On the other hand, we proved that left-handed children have better simple reaction times. A similar result was also found in a study conducted by Boulinguez et al., using the visual retention tests [19]. However, a study on right-handed split-brain patients has shown that those patients performed better when asked to copy simple line drawings with their left hands when compared with their dominant right hands, indicating that the right hemisphere has superior visuospatial functions [10]. This disagreement with our results could be attributed to the different tools that we used in our study.

Our study showed that reaction time tests which incorporated elements of visual memory gave the edge to right-handed children, whereas left-handed children proved to have better simple reaction times. This finding is in accordance with the majority of literature; the theory that the left hand is faster at reaction times that involve spatial relationships has been supported by the previous results [20–22]. Similarly, a study by Peters and Ivanoff showed that left-handed women react faster to auditory stimuli with their left hands than right-handed women do with their right hands, with no difference in reaction time to visual stimuli [23]. Dane and Erzurumluoglu [9] also found that left-handed handball players were faster than right-handed people when the left hand was tested, whereas no difference was observed between the two groups when the right hand was tested. Moreover, although right-handed male players had faster reaction times than their female counterparts, there was no difference in reaction times between left-handed male and female players. Griffith et al. [24] found that right-handed people were able to use a computer mouse faster with their right hand while left-handed people were equally fast with both hands.

Our study has some limitations. There were four different operators testing the students, with both the CANTAB and tests of handedness. While they read a uniform script of instructions, subtle differences may have occurred in the methods of delivering the tests. The touch screen used for the CANTAB test was pressure-sensitive which caused variation in responsiveness depending on individual subjects' strength. Finally, the cross-sectional nature of this study probably limited our ability to truly test the causality of the different factors associated with cognition.

## 5. Conclusion

To our knowledge, this is the first study to employ the CANTAB tool in assessing handedness and cognition in healthy children. There are some statistically significant though small differences in cognitive abilities between right- and left-handed individuals. Prospective studies are required to fully analyze the relationship between handedness and cognition. A larger sample size may have yielded more significant differences in cognition between right- and left-handed subjects. More in-depth investigation of the differences between right- and left-handed people can help lateralize certain cognitive functions, which can only serve to improve our understanding of the nature of handedness.

## Figures and Tables

**Table 1:** Correlation between measures of handedness and writing hand.

Variables	Writing hand
WBT^*∗*^	*r* = 0.595 *p* = 0.01

GPT^*∗∗*^	*r* = 0.487 *p* = 0.000

^*∗*^WBT: WatHand Cabient Test.

^*∗∗*^GPT: Grooved Pegboard Test.

**Table 2:** Handedness and confounders handedness and DMS^*∗*^.

	WatHand	GPT^*∗∗*^	Writing hand
Percent correct			
*r*	0.029	−0.090	0.060
*p*	0.761	0.350	0.535

Mean correct latency			
*r*	−0.118	−0.121	−0.038
*p*	0.221	0.207	0.695

Mean correct latency simultaneous			
*r*	−0.271	−0.120	−0.161
*p*	0.004	0.214	0.094

Errors correct color			
*r*	−0.067	0.081	−0.050
*p*	0.484	0.401	0.604

Errors correct shape			
*r*	−0.022	−0.030	−0.057
*p*	0.817	0.758	0.556

Errors novel distractor			
*r*	0.079	0.139	0.003
*p*	0.412	0.147	0.971

^*∗*^DMS: Delayed Matching to Sample.

^*∗∗*^GPT: Grooved Pegboard Test.

**Table 3:** Handedness and confounders handedness and PRM^*∗*^.

	WatHand	GPT^*∗∗*^	Writing hand
Percent correct			
*r*	0.144	0.205	0.216
*p*	0.139	0.034	0.025

Mean latency			
*r*	−0.009	−0.120	−0.047
*p*	0.928	0.217	0.633

^*∗*^PRM: Pattern Recognition Memory.

^*∗∗*^GPT: Grooved Pegboard Test.

**Table 4:** Correlation between handedness and RTI^*∗*^.

	WatHand	GPT^*∗∗*^	Writing hand
Mean simple reaction time			
*r*	−0.044	0.201	−0.077
*p*	0.648	0.036	0.422

Mean simple movement time			
*r*	−0.052	−0.002	−0.026
*p*	0.590	0.986	0.789

^*∗*^RTI: reaction time.

^*∗∗*^GPT: Grooved Pegboard Test.

**Table 5:** Correlation between handedness and SSP^*∗*^.

	WatHand	GPT^*∗∗*^	Writing hand
Span length			
*r*	−0.081	0.253	0.124
*p*	0.421	0.011	0.215

Total errors			
*r*	0.019	0.228	0.047
*p*	0.844	0.020	0.633

Total usage errors			
*r*	−0.235	−0.023	−0.132
*p*	0.016	0.816	0.180

^*∗*^SSP: Spatial Span.

^*∗∗*^GPT: Grooved Pegboard Test.
